# Microbial Biogeography along the Gastrointestinal Tract of a Wild Chinese Muntjac (*Muntiacus reevesi*)

**DOI:** 10.3390/microorganisms12081587

**Published:** 2024-08-04

**Authors:** Yuan Liu, Yan Shu, Yuling Huang, Jinchao Tan, Fengmei Wang, Lin Tang, Tingting Fang, Shibin Yuan, Le Wang

**Affiliations:** 1 Key Laboratory of Southwest China Wildlife Resources Conservation (Ministry of Education), China West Normal University, Nanchong 637009, China; liu0820_820@163.com (Y.L.); suyan99099@163.com (Y.S.); 19161036825@163.com (Y.H.); m18071927631@163.com (J.T.); 17323282261@163.com (F.W.); tanglin1004@163.com (L.T.); fangtingting_cwnu@163.com (T.F.); 2 Nanchong Key Laboratory of Wildlife Nutrition Ecology and Disease Control, Nanchong 637009, China

**Keywords:** Chinese muntjac, gut microbiota, gastrointestinal tract

## Abstract

The gut microbiota plays an important role in host nutrient absorption, immune function, and behavioral patterns. Much research on the gut microbiota of wildlife has focused on feces samples, so the microbial composition along the gastrointestinal tract of wildlife is not well reported. To address this gap, we performed high-throughput sequencing of 16s rRNA genes and ITs rRNA genes in the gastrointestinal contents of a wild adult male Chinese muntjac (*Muntiacus reevesi*) to comparatively analyze the microbial diversity of different gastrointestinal regions. The results showed that the dominant bacterial phyla were Firmicutes (66.19%) and Bacteroidetes (22.7%), while the dominant fungal phyla were Ascomycetes (72.81%). The highest bacterial diversity was found in the stomach, and the highest fungal diversity was found in the cecum. The microbial communities of the large intestine and small intestine were of similar structures, which were distinct from that of the stomach. These results would facilitate the continued exploration of the microbial composition and functional diversity of the gastrointestinal tract of wild Chinese muntjacs and provide a scientific basis for microbial resource conservation of more wildlife.

## 1. Introduction

The gastrointestinal tract of animals is a complex ecosystem composed of many microbes. The gut microbiota interacts with hosts to take in energy and nutrients while also affecting the host’s metabolism [1] behavioral patterns [2], immune function [3], and multiple other functions [4]. As confirmed by many studies, the composition and function of animal gut microbiota are affected by multiple factors such as species [5], diet [6], environment [7], and age [8]. For example, herbivores have higher microbial diversity compared to omnivores and carnivores [9]. Indeed, the intestinal microbial community profiles of foregut and hindgut fermenters are always significantly different [9]. In foregut fermenters (ruminant), microbial fermentation predominantly occurs in the rumen, whereas in hindgut fermenters, the cecum is the principal site for microbial fermentation [9]. Nowadays, studies of gut microbiota mainly focus on fecal samples as the end of the animal digestive tract [5,10,11]. However, the structure of the animal gut is complex, with significant differences in the structure, composition, and function of the microbiota in different intestinal segments. For example, a study in mice (*Mus musculus*) showed that the large intestine was enriched in anaerobic bacteria while the stomach and small intestine were enriched in parthenogenic anaerobic bacteria [12]. Furthermore, a study on wombats (*Phascolarctos cinereus*) also discovered both similarities and significant differences of the microbiota among the cecum, rectum, and fecal samples [13]. The microbial structure of sika deer (*Cervus nippon*) rectal contents and colon samples showed similarities and significant differences compared to that of small intestine contents [14]. Therefore, precise determination of microbial biogeography is of significance for deciphering the interaction between microbial symbionts and hosts.

Ruminants have a prominent place in the study of evolutionary biology and possess a high economic value [15]. Ruminants, which primarily feed on high-fiber plants, lack the ability to produce fiber-degrading enzymes [16]. Instead, they rely on intestinal microbial fermentation to generate volatile fatty acids, providing energy for their bodies [16]. Therefore, ruminants have a more diverse gut microbiota compared with other herbivores due to their feeding habits, enabling them to consume more substrates and increase food conversion efficiency [17]. Studies conducted on domesticated animals and captive wildlife indicate that ruminants have a higher diversity and abundance of gut microbes compared to monogastric animals [6,9]. As sequencing technology progresses and the research field broadens, the study of the composition and function of gut microbiota in wild ruminants has attracted increasing attention [18,19]. It is difficult to obtain intestinal content of wild animals, so stool samples have been extensively used; stool samples can be collected non-invasively and easily for most rare and threatened animals [18,19]. However, there are many differences between the microbial landscapes of feces and intestinal contents since fecal samples do not provide an accurate and complete picture of the gut’s microbial biogeographic composition [20]. The Chinese muntjac (*Muntjac reevesi*) is a small herbivore belonging to the genus Muntjac (*Muntiacus*) and mainly inhabits hills and open flat woodlands which are covered with diverse shrubs. It sustains itself primarily on a diet of wild fruits, seeds, and mushrooms and plays a pivotal role as a vector in the dispersal of plant seeds throughout subtropical regions [21]. As a protected wild animal in China, it is an important subject of scientific research and has economic value. We hypothesized that the different regions of the Chinese muntjac gut tract harbor distinct microbiota. Therefore, in this study, the contents of different intestinal segments of a wild muntjac were collected to investigate the structure of its gastrointestinal microbes with high-throughput sequencing technology. The primary objective was to explore the composition of the gastrointestinal microbes in the wild Chinese muntjac, laying the foundation for further investigations into the gut microbial compositions and functions of this species.

## 2. Materials and Methods

### 2.1. Ethics Statement

The research sample collection was the experiments were approved by the China West Normal University (No.2024LLSC0069).

### 2.2. Sample Collection

**Figure 1:**
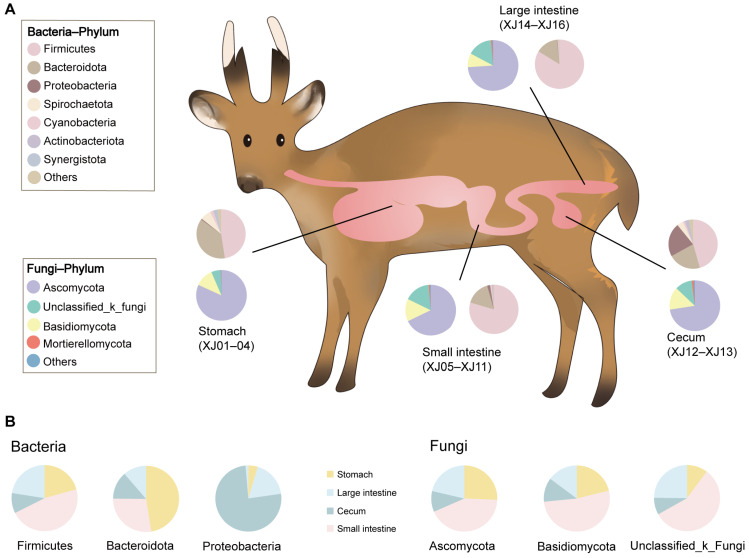
Diagram of gastrointestinal tract sampling of the Chinese muntjac and microbial composition. (**A**) Pie charts of bacterial and fungal phylum abundance in different in-testinal segments. (**B**) Pie chart of different intestinal segments in the bacterial and fungal phylum.

On 4 March 2022, a case of a wild male muntjac that had just died from predation by other wild animals (the body was still warm with blood in the internal organs not yet solidified) was found during field research in Foping National Nature Reserve in Shaanxi, which was subsequently collected and sent back to Sanguanmiao Conservation Station for an immediate autopsy. The contents of the stomach (rumen, reticular, omasum, and abomasum), small intestine (duodenum, jejunum, and ileum), cecum, and large intestine (rectum and colon) were collected sequentially in sterile sampling tubes along the intestinal segments for a total of 16 samples (Figure 1A). All samples were immediately placed in liquid nitrogen frozen, transported to the laboratory using dry ice, and stored at −80 °C until microbial DNA extraction.

### 2.3. DNA Extraction, PCR Amplification and Illumina MiSeq Sequencing

Gastrointestinal microbial DNA was extracted using the QIAamP PowerFecal ProDNA Kit (Qiagen, Hilden, Germany). DNA quality was examined using 1% agarose gel electrophoresis, and DNA concentration and purity were determined using NanoDrop2000.

The V3 and V4 hypervariable region of 16S rRNA genes was amplified using 338F (5′-ACTCCTACGGGAGGCAGCAG-3′) and 806R(5′-GGACTACHVGGGTWTCTAAT-3′) primers by an ABI GeneAmp^®^ 9700 PCR thermocycler (ABI, Foster city, CA, USA). The PCR amplification of the 16S rRNA gene was performed as follows: initial denaturation at 95 °C for 3 min, followed by 29 cycles of denaturing at 95 °C for 30 s, annealing at 55 °C for 30 s, and extension at 72 °C for 30 s, followed by single extension at 72 °C for 10 min, and ending at 4 °C. The PCR mixtures contain 5 × TransStart FastPfu buffer 4 μL, 2.5 mM dNTPs 2 μL, forward primer (5 μM) 0.8 μL, reverse primer (5 μM) 0.8 μL, TransStart FastPfu DNA Polymerase 0.4 μL, template DNA 10 ng, and finally ddH_2_O up to 20 μL. The ITS1 hypervariable region of ITS rRNA genes was amplified using ITS1F (5′-CTTGGTCATTTAGAGGAAGTAA-3′) and ITS2R (5′-GCTGCGTTCTTCATCGAT GC-3′) primers by an ABI GeneAmp^®^ 9700 PCR thermocycler (ABI, Foster city, CA, USA). The PCR amplification of the ITS rRNA gene was performed as follows: initial denaturation at 95 °C for 3 min, followed by 35 cycles of denaturing at 95 °C for 30 s, annealing at 55 °C for 30 s, extension at 72 °C for 30 s, and single extension at 72 °C for 10 min and finally at 4 °C. The PCR mixtures contain 5 × TransStart FastPfu buffer 4 μL, 2.5 mM dNTPs 2 μL, forward primer (5 μM) 0.8 μL, reverse primer (5 μM) 0.8 μL, TransStart FastPfu DNA Polymerase 0.4 μL, template DNA 10 ng, and finally ddH_2_O up to 20 μL. PCR reactions were performed in triplicate. Triplicate PCR reactions were conducted. The PCR products were then purified and quantified.Purified amplicons were pooled in equimolar amounts and paired-end sequenced on an Illumina MiSeq PE300 platform (Illumina, San Diego, CA, USA) according to the standard protocols by Majorbio Bio-Pharm Technology Co., Ltd. (Shanghai, China).

### 2.4. Data Analysis

The raw 16S rRNA gene and ITS rRNA gene sequencing reads were demultiplexed, quality-filtered by fastp version 0.20.0 [22], and merged by FLASH version 1.2.7 [23]. Denoising of the amplicon sequence variants (ASVs) was performed with the DADA2 QIIME2 plugin [24], and ASV clustering was performed by using 97% of the sequences consistently as the clustering criterion. Then, SILVA 99 database version 138 [25] and Unite ITS rRNA database [26] were used to assign taxonomic classification within QIIME2 via a Naïve Bayes sklearn classifier to classify ASVs at the genus level. 

The alpha diversity index was calculated using the vegan package of R version 4.3.1 after flattening the ASV data. The beta diversity was analyzed using the vegan package of R version 4.3.1, and a principal coordinate analysis (PCoA) was performed based on the Bray–Curtis distance, and significant differences between groups were analyzed using ANOSIM and PERMANOVA. All the significance levels were set at 0.05. We used the ggplot2 package of R version 4.3.1 for visualization of the analysis above.

## 3. Results

### 3.1. Bacterial Community Diversity

**Figure 2:**
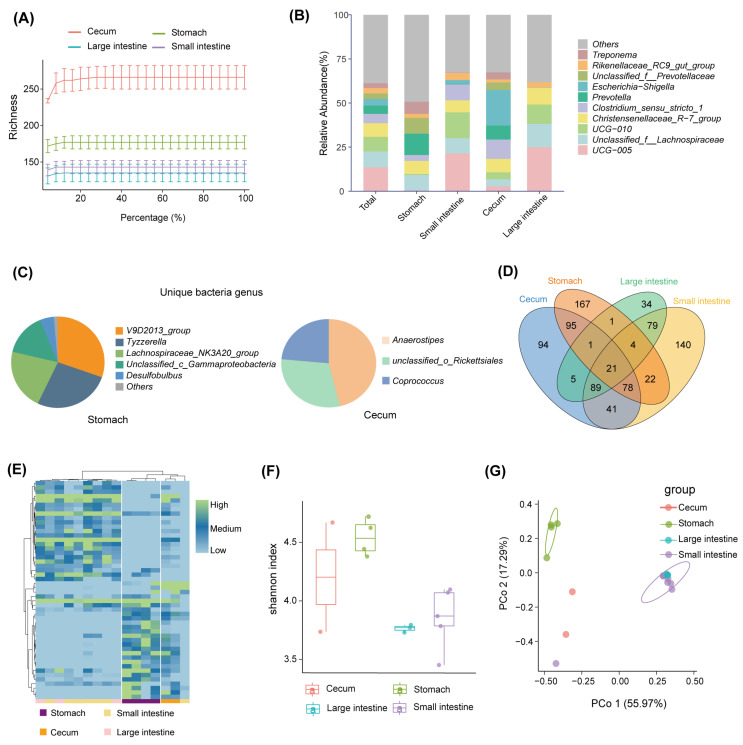
Bacterial diversity of the gastrointestinal tract. (**A**) Rarefaction curves based on Richness’s index. (**B**) The bacterial composition of the gastrointestinal tract at the genus level. (**C**) Unique bacterial genera of the gastrointestinal tract. (**D**) Venn diagrams of bacterial ASVs in the gastrointestinal tract. (**E**) Heat map of differential bacterial ASVs in the gastrointestinal tract. (**F**) Shannon index for bacterial ASVs in the gastrointestinal tract. (**G**) Principal coordinate analysis of bacterial ASVs in the gastrointestinal tract.

A total of 791,015 optimized sequences were obtained from the 16 samples, with an average sequence length of 299.67 base pairs (bp). By denoising the optimized sequences, 871 unique ASVs were obtained. The rarefaction curve of all samples tended to be flat, indicating that the sequencing data of all the samples were large enough, and the sequencing depth covered most species (Figure 2A). At the phylum level, the gastrointestinal bacteria were mainly seven phyla, including Firmicutes (66.19%), Bacteroidota (22.7%), Proteobacteria (4.17%), Spirochaetota (2.71%), Cyanobacteria (1.31%),Actinobacteriota (0.83%), and Synergistota(0.67%; see Figure 1A). The abundance of the bacterial phylum also differed across the gastrointestinal tract (Figure 1B). Firmicutes accounted for a large proportion of the small intestine (46.86%) and large intestine (22.52%) but a relatively low proportion of the stomach (20.75%) and cecum (9.87%); Bacteroides had the highest abundance in the stomach (47.47%) and a small portion in the intestine; and Proteobacteria were highly represented in the cecum (75.9%) and less common in other gastrointestinal tract regions (Figure 1B). At the genus level, UCG-005 (13.56%), unclassified_f_Lachnospiraceae (8.95%), UCG-010 (8.39%), Christensenellaceae_R-7_group (7.65%), and *Clostridium_sensu_stricto_1* (5.31%) were the dominant bacterial genera in the gastrointestinal tract (Figure 2B). The abundance of bacterial genera also differed along with the gastrointestinal regions. For example, *Prevotella* abundance was higher in the stomach and cecum and lower in the small intestine and large intestine, whereas the UCG-005 was dominant in the large intestine and small intestine (Figure 2B). In contrast, the most predominant genus in the cecum was *Escherichia-Shigella*, which was less prevalent in other intestinal segments (Figure 2B). All segments had different unique genera. For example, the V9D2013_group, *Tyzzerella*, and Lachnospiraceae_NK3A20_group were dominant unique genera in the stomach, whereas the *Anaerostipes*, unclassified_o_Rickettsiales, and *Coprococcus* were major unique genera in the cecum (Figure 2C).

The shared and unique ASVs in each gastrointestinal tract region were presented in a Venn diagram. The results showed that the four groups of samples shared a total of 21 ASVs, accounting for only 2.75% of the total number of ASVs (Figure 2D). Each intestinal segment had its unique ASVs, and the stomach had the maximum number of unique ASVs (167, accounting for 27.69% of all ASVs in the stomach; see Figure 2D). The abundance of differential ASVs across the gastrointestinal tract showed significant differences among different regions (Figure 2E). The stomach and the large intestine were of the highest and lowest bacterial diversity, respectively (Figure 2F). In addition, the PCoA plot based on the Bray–Curtis distance showed significant differences in the bacterial composition of the different segments at the level of ASVs. The small intestine and large intestine samples were significantly separated from the stomach (*p* < 0.05, Figure 2G).

### 3.2. Fungal Community Diversity

**Figure 3:**
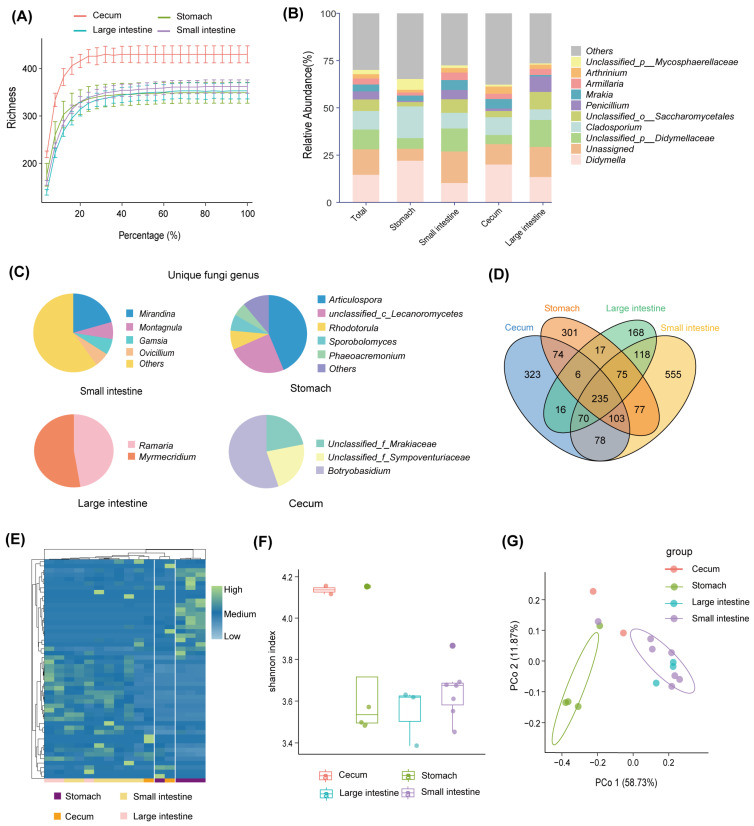
Fungal diversity of the gastrointestinal tract. (**A**) Rarefaction curves based on Richness’s index. (**B**) The fungal composition of the gastrointestinal tract at the genus level. (**C**) Unique fungal genera of the gastrointestinal tract. (**D**) Venn diagrams of fungal ASVs in the gastrointestinal tract. (**E**) Heat map of differential fungal ASVs in the gastrointestinal tract. (**F**) Shannon index for fungal ASVs in the gastrointestinal tract. (**G**) Principal coordinate analysis of fungal ASVs in the gastrointestinal tract.

A fungal data analysis yielded 816,975 optimized sequences with an average length of 239.27 bp. The optimized sequences with ASV sequences were denoised to obtain 2216 unique ASVs. The rarefaction curve of all samples became flat (Figure 3A). It suggested that the sequencing data of all samples were large enough, and the sequencing depth covered most species. At the phylum level, the gastrointestinal fungi were dominated by Ascomycota (72.81%), Unclassified_k_fungi (13.46%), Basidiomycota (12.59%), and Mortierellomycota (0.71%) (Figure 1A). The abundance of most fungal phylum also varied with the biogeography gastrointestinal tract (Figure 1B). Ascomycota was most common in the small intestine (42.84%). Unclassified_k_fungi were less abundant in the cecum (8.29%); Basidiomycota was enriched in the small intestine (52.24%) and less abundant in the cecum (11.72%) and large intestine (14.82%; see Figure 1B). At the genus level, Didymella (14.62%), Unassigned (13.46%), Unclassified_p_Didymellaceae (10.45%), and Cladosporium (9.82%) were the dominant fungal genera along the whole gastrointestinal tract (Figure 3B). The Didymella and Cladosporium were of the most abundant genera in the stomach; Unclassified_k_Fungi was the most abundant genus in the small intestine; Didymela was the dominant genus in the cecum; Unclassified_p_Didymellaceae was the most abundant genus in the large intestine, and low abundance was found in all the other sampled regions of the gastrointestinal tract (Figure 3B). In addition, the Articulospora, unclassified_c_Lecanoromycetes, and Rhodotorula were the most abundant unique genera in the stomach; Mirandina, Montagnula, and Gamsia dominated the unique genera of the small intestine; Unclassified_f_Mrakiaceae and Botryobasidium were the unique genera of the cecum; and Ramaria and Myrmecridium were the unique genera of the large intestine (Figure 3C).

There were 235 ASVs shared by different intestinal segments, accounting for 10.6% of the total number of ASVs (Figure 3D). In addition, all intestinal segments had their unique ASVs, of which the small intestine possessed the largest number of unique ASVs (555, accounting for 38.97% of the total number of ASVs in the small intestine; see Figure 3D). The heat map of differential ASV abundance showed significant differences among the different regions (Figure 3E). The highest fungal diversity was found in the cecum (Figure 3F). PCoA results showed marked differences in gastrointestinal fungi at the level of ASVs, which were similar to the bacterial community. The small intestine and large intestine samples were significantly separated from the stomach (*p* < 0.05, Figure 3G).

## 4. Discussion

The animal gastrointestinal tract is the site of ingested food digestion and absorption, and its physiological structure varies considerably between ruminants and monogastric animals. Monogastric animals have only one stomach, which is used for the digestion and decomposition of food [14], whereas ruminants have four stomachs (rumen, reticulum, omasum, and abomasum), and food is primarily fermented in the rumen and then enters the latter three stomachs for decomposition [14]. The microbiota in the gastrointestinal tract of ruminants plays a greater role in helping the digestive process compared to monogastric animals [27]. In this study, we focus for the first time on a wild ruminant, the Chinese muntjac, to investigate the composition and intergroup differences in the bacteria and fungi of its gastrointestinal tract (stomach, small intestine, cecum, and large intestine).

Many studies find that Firmicutes and Bacteroidota are widely present in the gut microbiota of deer families such as elk (*Cervus canadensis*) [28], roe deer (*Capreolus pygargus*) [29], and sika deer [30]. These findings are consistent with the dominant bacteria phylum observed in this study in the gastrointestinal tract of Chinese muntjac. We found that the abundance of Firmicutes was highest in the large intestine and small intestine. Its role is to break down cellulose in the food of herbivores and supply the host with energy [31], which is consistent with the results of a study conducted on the Sika deer [14]. Bacteroidota, which has the highest abundance in the stomach, is involved in soluble polysaccharide and protein degradation, enhancing the utilization and storage of nutrients in the host [32]. Then, some microbes are present in most regions but vary in abundance from region to region. For example, *Prevotella* has the highest abundance in the stomach and cecum and lower abundance in the small intestine and large intestine. This distribution was similar to that found in a goat (*Capra hircus*) study [33]. *Prevotella* can degrade multiple non-cellulosic polysaccharides and proteins, including hemicellulose, pectin, and starch [34]. The rumen is one of the main sites of fermentation of cellulose, hemicellulose, and pectin in ruminants, resulting in the production of significant amounts of short-chain fatty acids (SCFAs) [35]. However, the cecum is one of the main parts of hindgut fermentation in ruminants, which is able to ferment part of the digestible cellulose and produce a certain amount of SCFAs [35]. Therefore, these two regions have similarities in function and probably lead to the presence of part of the same microbiota. In addition, the UCG-005 has the highest proportion in the large intestine and small intestine. It is also the dominant bacterial genus in these regions, similar to the results found in goats [36]. UCG-005 has been shown to enhance the degradation of a large number of fibers in ruminants, which may be able to explain the enrichment of UCG-005 in the large intestine and small intestine [37]. Unique genera were found in most gastrointestinal tract regions except for the large intestine and small intestine. The large intestine plays key roles in the culminating phase of the processing of nutrients, where excess water from the undigested remnants is absorbed and waste materials are compacted into feces for eventual excretion [38]. Compared to the stomach, the large intestine processes less digesta, which may explain the absence of a unique genus of bacteria in the large intestine. The small intestine is much longer than other intestinal segments and contains high concentrations of bile salts and digestive enzymes, making it difficult for bacteria to grow [39]. Stomach and cecum possess the unique microbiota that plays an exclusive role. For example, the V9D2013_group, a unique bacterial genus in the stomach, is a genus capable of producing butyrate, which inhibits inflammation and oxidative stress [40]. *Anaerostipes* is also a genus capable of producing butyrate and is unique to the cecum [41]. This suggests that the unique bacterial genus in the gastrointestinal tract of Chinese muntjac may be related to the production of SCFAs. In addition, Ascomycota, the dominant fungal phylum widely found in the gastrointestinal tract, is present not only in ruminants but also in monogastric animals [42]. Both *Didymella* and *Cladosporium* belong to Ascomycota and play an essential role in the digestion of fibrous plants ingested by herbivores [43]. However, all the results suggested that there were substantial differences in the microbial composition of the gastrointestinal tract, but further studies are needed to verify whether these microbiotas represent functional and metabolic differences.

In our study, the proportions of shared bacterial and fungal ASVs were 2.4% (21/871) and 10.6% (235/2216), respectively. These results indicate that the similarity of bacteria in the gastrointestinal tract of Chinese muntjac is lower than that of fungi. Shared ASVs usually have similar metabolic pathways and functions between different regions [44]. All gastrointestinal regions have their unique ASVs, with the stomach having the most bacteria-unique ASVs and the small intestine having the most fungal unique ASVs. The rumen of ruminants is a natural fermenter for the degradation of fibrous substances, colonized by a variety of microorganisms that digest and utilize food, so the stomach has the most unique ASVs [27]. Additionally, the food consumed by ruminants stay in the stomach for an extended period, leading to increased colonization and proliferation time of a large number of bacteria [45]. The small intestine can ferment monosaccharides and amino acids, which is suitable for the growth of most anaerobic fungi and can efficiently utilize a portion of simple carbohydrates [12,46]. This may explain why the small intestine has the greatest number of unique fungal ASVs. Our results showed higher diversity in stomach and cecum samples and lower diversity in small and large intestine samples, indicating the regional specialization of gut microbiota which also been found in multiple species of different classes [12,42]. On the one hand, different physical and chemical conditions, such as the pH value, oxygen concentration, morphological structure, and nutrient availability, are all associated with microbiota diversity [47]. On the other hand, herbivores tend to consume large amounts of high-fiber plants, and the stomach becomes the first site through which food passes on the distal gastrointestinal tract. Thus, it is enriched by a variety of microbiota responsible for digestion and decomposition of various plant fiber components, with a tendency toward higher bacterial diversity [45]. In the meanwhile, as an important fermentation organ, the cecum can provide a large number of SCFAs for ruminants through microbial degradation of structural carbohydrates such as cellulose and hemicellulose [48]. Thus, the higher fungal diversity in the cecum may be associated with energy production in the Chinese muntjac. The microbial communities of the small intestine and large intestine significantly overlapped and were different from those of the stomach, suggesting that the microbial structure of the stomach is different from that of the small and large intestine. The main role of the stomach is to break down food, but the intestine is the main place to absorb food nutrients [49]. So, the differences of microbial community between stomach and intestine are consistent with the functional differentiation of the stomach and intestine.

## 5. Conclusions

This study reported for the first time the structure and diversity of bacterial and fungal communities in the gastrointestinal tract of Chinese muntjac and revealed markedly biogeographic heterogeneity at both the fungal and bacterial levels, with significant differences between the stomach and intestinal microbiota. Since the sample for this study was obtained opportunistically from the wild, more samples need to be collected to summarize gut microbes’ composition and functional diversity in Chinese muntjac populations. Alternatively, further research can be conducted in captive settings. Furthermore, the functional metabolism of microbiota in each gut segment needs to be verified by integrating metagenome and metabolomic methods. 

## Data Availability

This study’s raw sequence data was submitted to the Genome Sequence Archive database with accession number CRA012081.
